# Tribological Properties of Groove-Textured Ti-6Al-4V Alloys with Solid Lubricants in Dry Sliding against GCr15 Steel Balls

**DOI:** 10.3390/mi14111978

**Published:** 2023-10-25

**Authors:** Ze Wu, Xiuli Tan, Guochao Li, Youqiang Xing

**Affiliations:** 1 School of Mechanical Engineering, Southeast University, Nanjing 211189, Chinayqxing@seu.edu.cn (Y.X.); 2 School of Mechanical Engineering, Jiangsu University of Science and Technology, Zhenjiang 212100, China

**Keywords:** textured groove, lubrication, Ti-6Al-4V alloy, friction

## Abstract

A nanosecond laser is used to fabricate groove-patterned textures on the upper surface of Ti-6Al-4V alloys, and then molybdic sulfide solid lubricants are filled into the grooves. The treated titanium alloy is subjected to friction and wear tests. The tribological performances of Ti-6Al-4V alloys are evaluated, and the wearing mechanism is analyzed. The combination of solid lubricants and surface texturing can effectively reduce the frictional coefficient and reduce the adhesion of Ti-6Al-4V materials on the steel balls for friction. The main wearing mechanism is the adhesive wear of the Ti-6Al-4V alloy and the adhesion of Ti-6Al-4V alloy materials on the surface of the steel balls. During the friction process, solid lubricants are squeezed from the grooves and coated at the friction interface to form a solid lubrication layer. This is the important reason why the combination of surface texturing and solid lubricants can improve the friction properties of titanium alloys effectively. The combination of solid lubricants and laser surface texturing provides an effective alternative way to improve the tribological properties of titanium alloy materials.

## 1. Introduction

Titanium alloys are widely used in engineering fields, including in energy components, aerospace, and automotive engineering, because of their low density, high specific strength, and corrosion resistance [1,2,3]. Among the numerous titanium alloy materials, the Ti-6Al-4V titanium alloy is the most widely used. However, its low hardness and low wear resistance limit its high performance in engineering applications [4,5,6,7]. Surface modification can effectively improve the tribological properties of titanium alloys. In an effort to improve the wear resistance of titanium alloy surfaces, some surface modification technologies such as coating technology, surface laser cladding, new lubricating materials, and surface texturing have been proposed, and these methods have achieved good results to a certain extent [8,9,10,11,12].

Surface coating is the most commonly used method to improve the tribological performances of titanium alloys [13,14,15]. Bian et al. [16] proposed a coating with a Cu layer and high-hardness TiN layer deposited on the Ti-6Al-4V titanium alloy, and this coating had both wear-resistant and antifriction properties. It was also confirmed by Burkov et al. [17] that electrospark Cu-Ti coatings with a wide range of titanium and copper ratios had excellent corrosion and wear resistance values for the Ti-6Al-4V titanium alloy. It was reported by Yu et al. [18] that (ZrTaNb)C/N quaternary ceramic coatings prepared via dual-cathode glow plasma surface alloying can improve the surface hardness and wear resistance of aviation titanium alloys. In addition to surface coatings, the development of some new lubricants has also provided new avenues for improving the tribological properties of titanium alloys. It was found by Yang et al. [19] that nonylphenol polyoxyethylene ether phosphate ester solution can decrease the friction coefficient and wear rate of titanium alloys against tungsten carbide. It was reported by Chen et al. [20] that a lubricating layer can be cemented onto the Ti-6Al-4V surface by mimicking the adhesion proteins secreted by mussels in the ocean; as a result, the generation and disappearance of hydration layer can be controlled by utilizing the photothermal conversion ability of molybdenum disulfide nanosheets to near-infrared light. Laser surface texturing is also an effective means of improving the tribological properties of structural components [21]. Pan et al. [22] indicated that femtosecond laser-induced surface modification can improve the anti-friction properties of Ti-6Al-4V titanium alloys. It was reported by Schneider et al. [23] that finishing procedure, packing density, and atmosphere during laser texturing were varied between the experiments when linear textures on Ti-6Al-4V alloys were fabricated by a nanosecond laser. Mukherjee et al. [24] indicated that different process parameters had effects on the microstructural and bio-interfacial aspects of the biocompatibility of titanium alloys. It was also reported by Grabowski et al. [25] that a new microstructure, as well as its surface’s topography on Ti-6Al-4V alloys, was obtained via the rapid solidification of surface lasering.

In our previous studies, we investigated the effect of textured dimples on the tribological performance of cemented carbide [26]. In the present study, a method combining laser surface texturing with solid lubricants is proposed. For this study, groove-patterned textures were fabricated on the surface of the titanium alloys by a nanosecond laser and filled with solid lubricants. The treated titanium alloy was subjected to friction and wear tests. The tribological properties of the titanium alloys were evaluated through using scanning electron microscopy (SEM), stereoscopic microscopy, and white light interferometry. The wear mechanism of the titanium alloys was also analyzed. The novelty of the present work lies in the fact that it describes how to prepare textured grooves on the surface of Ti-6Al-4V alloys, which is different from our previous study about textured dimples on cemented carbide. This method of combining laser surface texturing with solid lubricants can be used as an alternative method to improve the tribological properties of titanium alloys, expanding the engineering applications of titanium alloy materials.

## 2. Experiment

### 2.1. Materials for Friction

The used specimens were Ti-6Al-4V alloy discs. The diameter of each disc was 60 mm, while the thickness was 4 mm. The composition of the Ti-6Al-4V alloys consisted of 6 wt% aluminum element and 4 wt% vanadium element. The hardness of all Ti-6Al-4V alloys was 36 HRC, and their compressive yield strength was 970 MPa. The selected specimens for friction were GCr15 steel balls with a diameter of 9.5 mm. The composition of the GCr15 steel consisted of 1.5 wt% chromium element, 0.25 wt% molybdenum element, and 0.3 wt% manganese element. The hardness of the GCr15 steel was 60 HRC.

### 2.2. Fabrication of Textured Grooves

**Figure 1:**
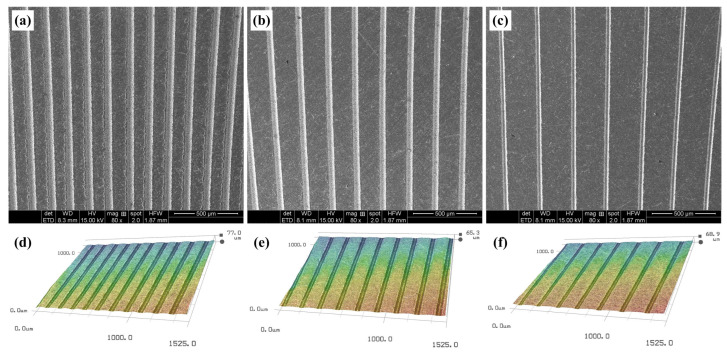
SEM morphologies of different textured grooves—(**a**) TG150, (**b**) TG200, and (**c**) TG250—and the corresponding three-dimensional morphologies of (**d**) 3D TG150, (**e**) 3D TG200, and (**f**) 3D TG250.

**Figure 2:**
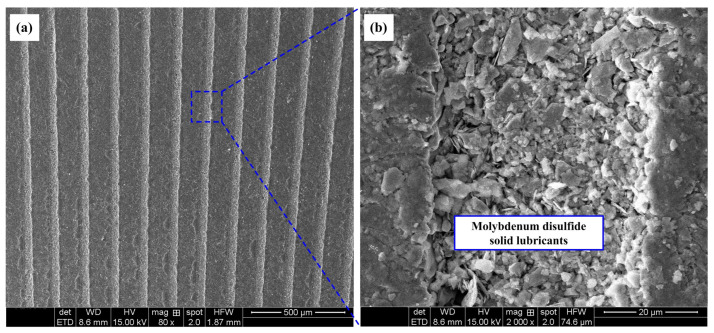
Micro topographies of textured grooves filled with molybdenum disulfide solid lubricants: (**a**) TG200 filled with MoS_2_ and (**b**) the corresponding magnified view of a single groove.

The upper surfaces of the Ti-6Al-4V alloy discs were polished using an automatic polishing machine to obtain a smooth surface roughness (*Ra* ≤ 0.05 μm). Groove-patterned textures were then generated on the upper surface of the Ti-6Al-4V alloy discs by using a nanosecond laser. The laser pulse was generated using a Nd: YAG laser system at a pulse duration of about 20 ns, repetition rate of 2 kHz, and center wavelength of 1064 nm. Machining was accomplished in air with an average working current of 12 A and an operating voltage of 10 V. The working temperature of the laser process was room temperature. After the laser process, burrs around the edge of the grooves were removed via manual polishing. The textured grooves were observed using a 3D microscope (Keyence VHX-2000). The width of a single groove was about 30 μm, and the depth was about 70 μm. Three kinds of textured patterns with groove distances of 150 μm (TG150), 200 μm (TG200), and 250 μm (TG250) were designed and fabricated, respectively. Due to the instability of the laser processing, there were slight differences in the width of the textured grooves among the different samples, but the width of the individual grooves in all samples was around 30 μm. The textured patterns with different groove distance values on the surface of titanium alloy are shown in Figure 1a–c, while the corresponding stereoscopic topographies are presented in Figure 1d–f. Some of the textured samples were selected to fill molybdenum disulfide (MoS_2_) solid lubricants in the textured grooves. Under microscope observation, molybdenum disulfide solid lubricants with a particle size below 2 μm were filled into the grooves. Firstly, the solid lubricants were smeared on the textured surface, and then the solid lubricants were pressed into the grooves using a fine needle. Sufficient solid lubricants were smeared on the textured surface, and then the solid lubricants were repeatedly pressed by the needle tip to ensure that all the texture grooves were filled with solid lubricants and compacted without further filling. The SEM morphologies of the textured grooves filled with MoS_2_ are shown in Figure 2.

### 2.3. Friction and Wear Test

**Figure 3:**
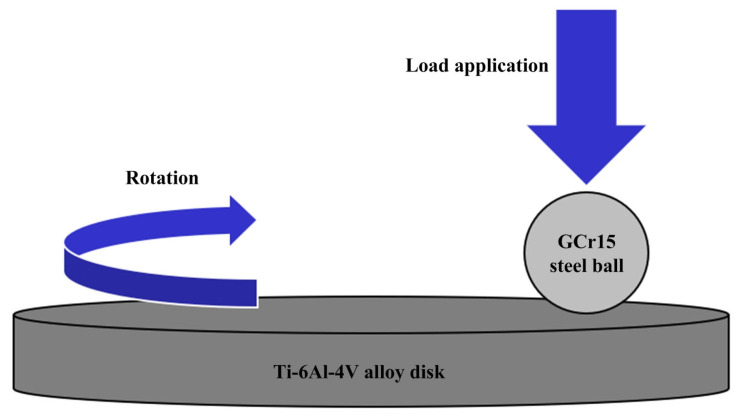
Schematic diagram of the friction and wear test.

A rotating ball-on-disc friction and wear test was conducted on a friction testing machine (UMT-2), and the tribological properties of the groove-textured titanium alloys when sliding against the GCr15 steel balls were studied. A schematic diagram of the friction and wear test can be seen in Figure 3. The titanium alloy disc rotates continuously at high speed, while the steel ball is fixed by a special fixture. The sliding speeds for conducting the friction and wear tests were set to 20 m/min, 40 m/min, 60 m/min, 80 m/min, and 100 m/min; the loaded load was 10 N, and the sliding time for a single test was 10 min. The nominal contact pressure was 420 MPa. The smooth sample (SS), i.e., the sample that was not subjected to laser texturing, and the textured samples with and without the molybdenum disulfide solid lubricants were tested for comparison.

The friction coefficients for the different friction tests were compared. The topographies of the worn titanium alloy discs and the GCr15 steel balls were investigated via scanning electron microscopy (SEM), white light interferometry (Wyko NT9300), and X-ray energy spectrum analysis (EDS). The wear mechanism of the titanium alloys was also analyzed.

## 3. Results and Discussion

### 3.1. Friction Coefficients

**Figure 4:**
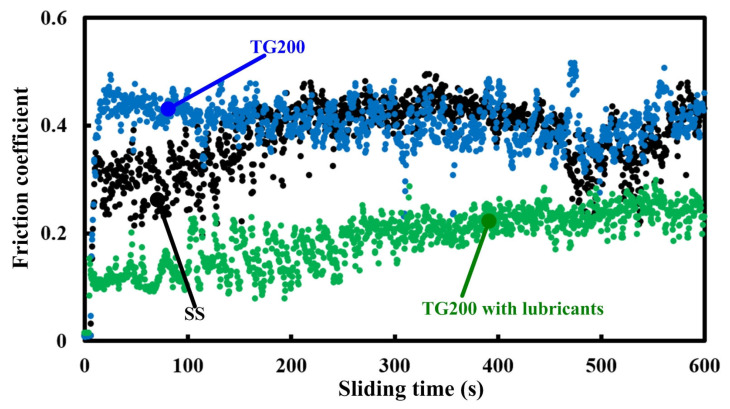
The variation trends of the friction coefficients with sliding time for the different samples at a sliding speed of 20 m/min.

The variation trends in the friction coefficients of the SS, TG200, and TG200 with lubricants at a sliding speed of 20 m/min are shown in Figure 4. It can be seen that the coefficient for the TG200 sample has values of a similar size compared to that of the SS. However, even at the beginning of the friction process, the TG200 sample has a higher friction coefficient compared to the SS. In other words, a single-groove-patterned texture on the upper surface of the Ti-6Al-4V alloy does not reduce the friction coefficient. Compared to the SS and TG200 sample, the TG200 sample with lubricants can obtain a significantly smaller friction coefficient. For all samples, there are significant fluctuations in the friction coefficient. It is worth mentioning that the fluctuation in the friction coefficients obtained by the TG200 sample is the largest.

**Figure 5:**
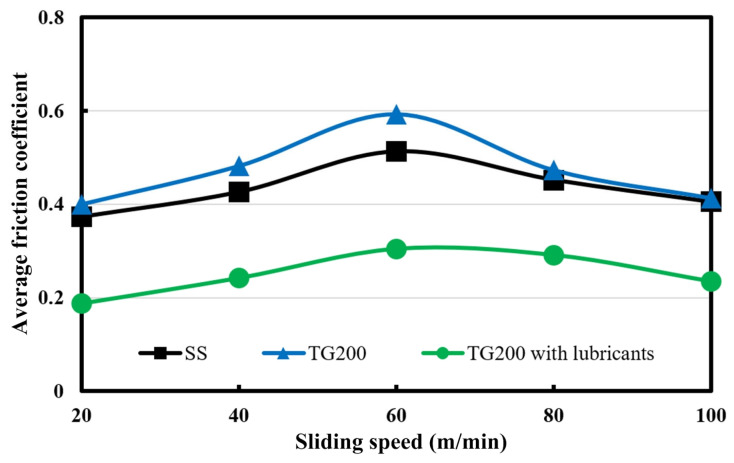
The average friction coefficient varies with sliding speed for different samples.

The friction coefficients of each set of tests were calculated, i.e., the average friction coefficients were calculated. The average friction coefficients for the SS, the TG200 sample, and the TG200 sample filled with MoS_2_ at different frictional speeds are indicated in Figure 5. Overall, the friction coefficient first increases and then decreases with the increase in sliding speed. Under the same condition, the friction coefficient of TG200 sample is slightly higher than that of the SS, while the TG200 sample with lubricants has a significantly lower average friction coefficient. The combination of surface texturing and solid lubricants exhibits significant friction reduction effects.

**Figure 6:**
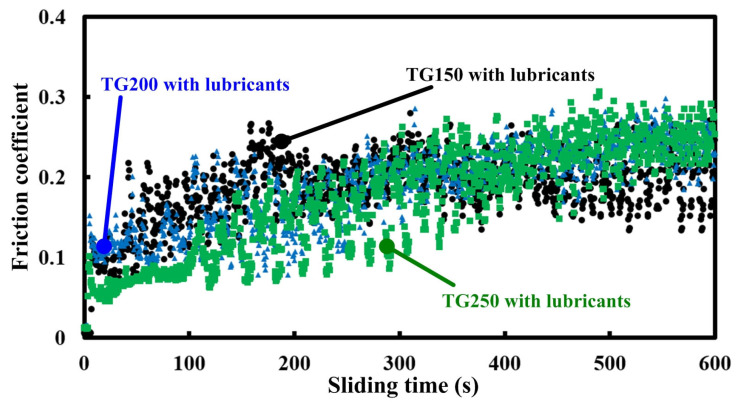
Changing trends of friction coefficients for different-density textured groove samples at a frictional speed of 20 m/min.

**Figure 7:**
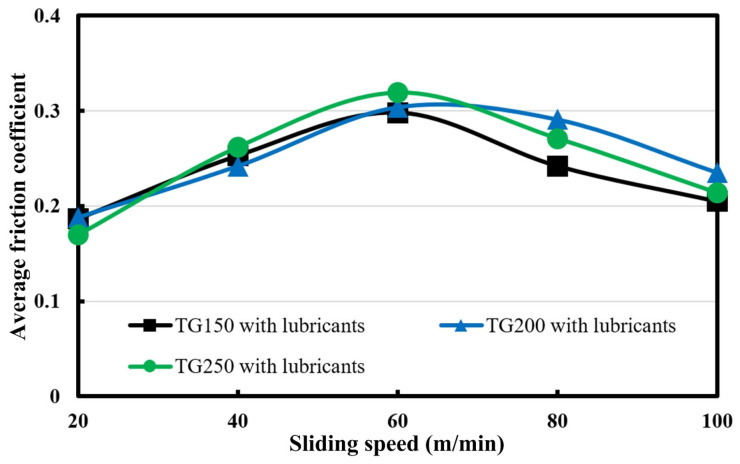
The average friction coefficient varies with frictional speed for the different textured groove samples.

Through the above analysis, it can be found that the combination of surface texturing and solid lubricants can effectively reduce the friction coefficients for titanium alloys. The changing trends regarding the friction coefficients for different-density textured groove samples at a frictional speed of 20 m/min are shown in Figure 6. It can be seen that as the sliding time increases, the friction coefficients of all the samples show a slow increasing trend. In addition, samples with different groove densities did not show any differences in friction coefficient values. Figure 7 indicates the average friction coefficients for different textured groove samples with solid lubricants at different frictional speeds. Overall, as the sliding speed increases, the friction coefficients of all specimens also show a trend—first increasing and then decreasing. Under the same conditions, there is no obvious difference in the average friction coefficients generated by samples with different groove densities. That is to say, within the experimental conditions, the difference in groove density does not have a significant impact on the friction coefficient.

### 3.2. Worn Surface of the Steel Balls and the Titanium Alloy Discs

**Figure 8:**
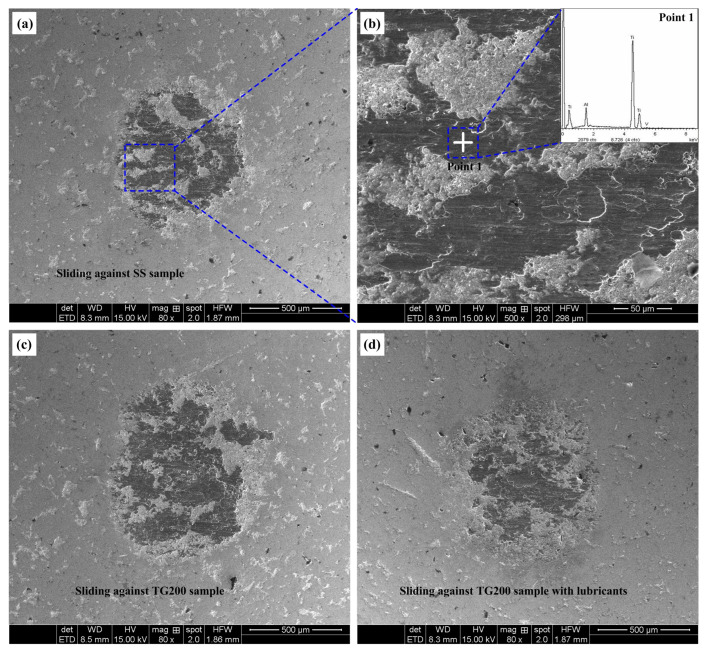
Wear morphologies of the worn surfaces of the GCr15 steel balls rubbing against (**a**) the SS ((**b**) enlarged view of selected worn area), (**c**) the TG200 sample, and (**d**) the TG200 sample with molybednum disulfide at the speed of 20 m/min after 10 min sliding friction.

Wear is an important factor in evaluating the tribological properties of materials. The worn surfaces of the titanium alloys and the steel balls were investigated. The worn surfaces of the GC15 steel balls sliding against the SS, the TG200 sample, and the TG200 sample with molybdenum disulfide at a frictional speed of 20 m/min after 10 min sliding friction are shown in Figure 8. It can be seen from Figure 8 that all steel balls have titanium alloy materials adhered to their surfaces. The wear area on the surface of the steel ball rubbing against the TG200 sample is significantly larger than that of the smooth sample and the TG200 sample with molybdenum disulfide. Compared to the smooth sample, the TG200 sample with lubricants can reduce the adhesion of the titanium alloy materials to the surface of the steel balls. That is to say, a single surface texturing exacerbates the wear state of the surface of the steel balls, and only the combination of surface texturing and solid lubricants can reduce the wear of the steel balls.

**Figure 9:**
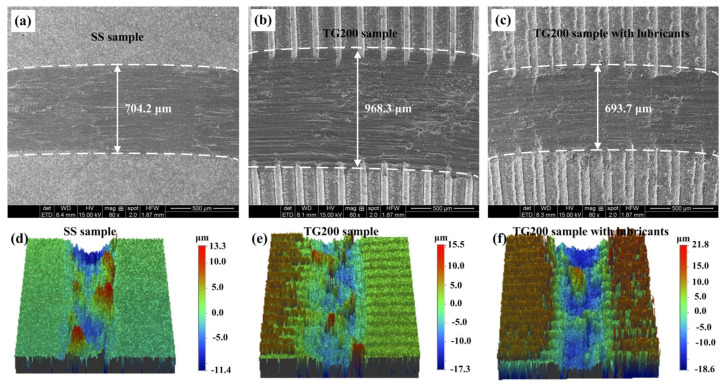
SEM images of the worn surfaces of (**a**) the SS, (**b**) the TG200 sample, and (**c**) the TG200 sample with lubricants after 10 min of dry operation at the speed of 20 m/min; (**d**) 3D worn surface of the SS, (**e**) 3D worn surface of the TG200 sample, (**f**) 3D worn surface of the TG200 sample with lubricants.

The worn surfaces of the titanium alloy were also investigated. The wear morphologies of the worn surfaces of the SS, the TG200 sample, and the TG200 sample with molybdenum disulfide at the speed of 20 m/min after 10 min sliding friction are shown in Figure 9. It can be seen from Figure 9 that the width of the wear marks on the SS, the TG200 sample, and the TG200 sample with lubricants were 704.2 μm, 968.3 μm, and 693.7 μm, respectively. That is to say, the width of the wear marks on the TG200 sample was significantly wider compared to that on the smooth sample. However, the width of the wear marks on the TG200 sample with lubricants was similar to that on the smooth sample. The 3D morphologies of the wear marks (Figure 9d–f) indicate that there is no significant difference in the depth of the wear marks between the different samples. That is to say, single groove texturing increases the width of the wear marks on the titanium alloys, and the combination of groove texturing and solid lubricants does not significantly reduce the wear of the titanium alloys. Under the experimental detection conditions, no significant wear loss in weight for the titanium alloy samples was found. The wear marks were mainly due to the plastic deformation of the titanium alloy materials, which was caused by the extrusion from the friction process.

### 3.3. Discussion

From the above analysis, it can be seen that a single surface texturing does not reduce the friction coefficient; instead, it increases the wear of the titanium alloys and the steel balls. Only the combination of surface texturing and solid lubricants can significantly reduce the friction coefficient and reduce the adhesion of the titanium alloy materials on the surface of the steel balls. During the entire friction process, due to the difference in hardness between the titanium alloy materials and the steel balls, no detachment of hard-phase wear particles was found. The wear mechanism was mainly the adhesive wear of the titanium alloy materials and the adhesion of the titanium alloy materials on the surface of the steel balls. In general, as the sliding speed increases, the friction between the titanium alloys and the steel balls becomes severe and the friction coefficient increases. However, an increase in sliding speed will generate more heat, which would cause the titanium alloy material to soften and result in a decrease in friction coefficient [27]. This is precisely why, in the present study, the average friction coefficient initially increases and then reduces.

**Figure 10:**
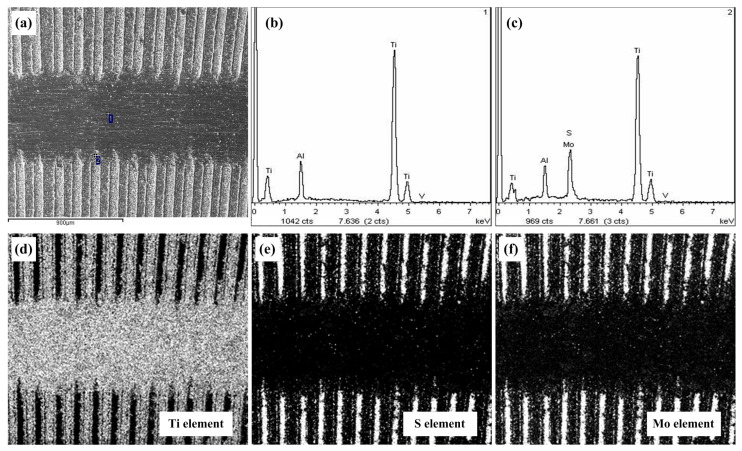
Wear morphology and component analysis for the TG150 sample filled with molybednum disulfide at a frictional speed of 20 m/min: (**a**) SEM image, (**b**) XRD for point 1, (**c**) XRD for point 2, (**d**) EDS distribution of Ti element, (**e**) EDS distribution of S element, and (**f**) EDS distribution of Mo element.

The wear morphology and component analysis for the TG150 sample filled with molybdenum disulfide at a frictional speed of 20 m/min are shown in Figure 10. It can be seen that the molybdenum disulfide solid lubricants are stored in the grooves. The titanium alloy in the area of the wear marks is squeezed and filled into grooves. However, in the area of the wear marks, except for the titanium alloy materials, a small amount of molybdenum disulfide solid lubricants can still be found. In fact, during the friction process, the solid lubricants stored in the grooves are squeezed and coated at the friction interface, forming a dynamic lubrication layer. This is the key reason why the combination of surface texturing and solid lubricants can reduce the friction coefficient and reduce the adhesion of titanium alloy materials on the surface of steel balls. However, a single surface texturing does not reduce the friction coefficient, mainly because there is no lubricating medium. The increase in surface roughness was caused by the presence of the textured grooves and the scraping of the groove edges, which are the important reasons behind the increased wear of the titanium alloy materials.

## 4. Conclusions

A method combining laser surface texturing with solid lubricants has been proposed to improve the tribological performances of titanium alloy materials. The groove-patterned textures were fabricated on the surface of Ti-6Al-4V alloys using a nanosecond laser and filled with molybdenum disulfide solid lubricants. The friction coefficients based on different friction tests were compared. The topography of the worn titanium alloy discs and the GCr15 steel balls were investigated using scanning electron microscopy (SEM), white light interferometry (Wyko NT9300), and energy dispersive X-ray analysis (EDS). The wear mechanism of titanium alloys was also analyzed. A single surface texturing did not reduce the average friction coefficient, but it did increase the wear of the titanium alloys due to the presence of the grooves. Only the combination of surface texturing and solid lubricants could effectively reduce the average friction coefficient and reduce the adhesion of the titanium alloy materials on the surface of the steel balls. The difference in the densities of the textured grooves did not significantly affect the friction performance of the titanium alloys. During the entire friction process, due to the difference in hardness between the titanium alloy materials and the steel balls, no detachment of hard-phase wear particles was found. The main wear mechanism was the adhesive wear of the titanium alloys and the adhesion of the titanium alloy materials on the surface of the steel balls. During the friction process, solid lubricants are squeezed from the grooves and coated at the friction interface to form a solid lubrication layer. This is the important reason why the combination of surface texturing and lubricants can effectively improve the friction performance of titanium alloys. The combination of laser surface texturing and solid lubricants provides an effective alternative way to improve the tribological performances of titanium alloy materials, which will have a positive impact on promoting the engineering applications of titanium alloy materials.

## Data Availability

The data that support the findings of this study are available on request from the corresponding author.
